# Impact of dietary forage proportion and crossbreeding on feed efficiency and methane emissions in lactating dairy cows

**DOI:** 10.1016/j.aninu.2024.08.011

**Published:** 2024-12-28

**Authors:** Sabrina Ormston, Tianhai Yan, Xianjiang Chen, Alan W. Gordon, Katerina Theodoridou, Sharon Huws, Sokratis Stergiadis

**Affiliations:** a Department of Animal Sciences, School of Agriculture, Policy and Development, University of Reading, Reading RG6 6EU, United Kingdom; b Agri-Food and Biosciences Institute, Hillsborough, Co. Down BT26 6DR, United Kingdom; c Agri-Food and Biosciences Institute, Statistical Services Branch, Newforge Lane, Belfast, Co. Antrim BT9 5PX, United Kingdom; d Institute for Global Food Security, Queen's University Belfast, Belfast BT9 5DL, United Kingdom

**Keywords:** Breed, Feed efficiency, Holstein-Friesian, Jersey, Methane, Norwegian Red

## Abstract

Increasing forage proportion (FP) in the diets of dairy cows would reduce competition for human edible foods and reduce feed costs, particularly in low-input systems. However, increasing FP reduces productivity and may increases methane (CH_4_) emission parameters. This work aimed to investigate the impact of FP and breed on feed efficiency and CH_4_ emission parameters. Data from 32 individual experiments conducted at the Agri-Food and Biosciences Institute between 1992 and 2010 were utilised in this study resulting in data from 796 Holstein-Friesian (HF), 50 Norwegian Red (NR), 46 Jersey × HF (J × HF) and 16 NR × HF cows. Diets consisted of varying proportions of forage and concentrate dependent on the experimental protocols of each experiment. A linear mixed model was used to investigate the effect of low (LFP; 10% to 30%), medium (MFP; 30% to 59%), high (HFP; 60% to 87%) and pure (FOR; 100%) FP (dry matter [DM] basis) and breed on feed efficiency, and CH_4_ emission parameters and multivariate redundancy analysis identified associations between animal and dietary drivers on the same variables. Total dry matter intake (DMI) was higher for cows offered LFP (17.3 kg/d) and MFP (17.9 kg/d) compared to HFP (15.3 kg/d) and FOR (13.8 kg/d) (*P* < 0.001). Milk yield (*P* < 0.001), milk yield/DMI (*P* < 0.001), energy corrected milk (ECM)/DMI (*P* < 0.001) and milk energy/DMI (*P* < 0.001) were higher for LFP and MFP compared to HFP and FOR. Methane/DMI was higher for HFP (24.3 g/kg) compared to MFP (22.4 g/kg) (*P* < 0.001). Methane/milk yield (*P* < 0.001) or CH_4_/ECM (*P* < 0.001) was higher for HFP (22.5 or 21.6 g/kg) and FOR (27.0 or 25.8 g/kg) compared to MFP (19.1 or 17.9 g/kg). There were no differences between LFP and MFP or between HFP and FOR for milk yield, milk yield/DMI, ECM/DMI, milk energy/DMI, CH_4_/milk yield and CH_4_/ECM (*P* > 0.05). Differences existed between breeds for residual feed intake (*P* = 0.040), milk yield/DMI (*P* = 0.041) and CH_4_/DMI (*P* = 0.048) with multivariate redundancy analysis demonstrating negative correlations with efficiency and positive correlations with CH_4_/DMI and CH_4_/milk yield. Feeding concentrates at 70% to 90% of DMI (LFP group) would not result in any further benefits for productivity, feed efficiency or CH_4_ yield and intensity when compared to feeding 41% to 70% concentrates of DMI (MFP group). There may be opportunity to improve profitability for lower intensity farms with less concentrate input.

## 1 Introduction

The concept of increasing forage proportion (FP) in UK dairy diets has been receiving increased attention due to the increased costs, price volatility, and concerns around food-feed competition and environmental footprint associated with certain concentrate feeds (Bijttebier et al., 2017; Gaudaré et al., 2021). Although, it is well understood that reducing concentrate inclusion in dairy cow diets may result in decreased animal productivity (Agle et al., 2010; Broderick, 2003; Moorby et al., 2006; Yang and Beauchemin, 2007a). Approximately 60% of the costs associated with milk production are related to feeding (Connor, 2015) and concentrate feeding in particular partly represents food-feed competition (Garnsworthy and Wilkinson, 2017). As world population rises and demand for resources increases, ensuring the productivity and profitability of dairy systems, with reduced environmental footprint, will be essential (Brito et al., 2021). Whilst, it is well known that supplementing diets with higher energy concentrates results in higher productivity (Agle et al., 2010; Yang and Beauchemin, 2007a) and often improved feeding efficiency (Agle et al., 2010; Broderick, 2003), reducing reliance on concentrate feeding by increasing the FP in dairy cow diets would reduce food-feed competition and cost of production (Gaudaré et al., 2021); traits which are highly relevant to lower input and pasture-based dairy farming (Bijttebier et al., 2017). In such conditions, ensuring that a high forage-to-milk conversion is achieved is also key to a farms’ profitability (Hayes et al., 2013). In many cases, increases in milk yield are primarily a result of increased dry matter intake (DMI) (Lawrence et al., 2015; Moorby et al., 2006) and thus in these cases milk yield/DMI may be similar between different FP (Moorby et al., 2006). It has been suggested that DMI is a stronger driver for feed efficiency than milk yield (Ben Meir et al., 2021) and studies which have found improved milk yield/DMI following a reduction in FP or neutral detergent fibre (NDF), have attributed this to higher milk yields without simultaneous increases in DMI (Agle et al., 2010; Broderick et al., 2009). Therefore, a moderate increase in FP may positively impact feed efficiency in some occasions by reducing DMI (Ben Meir et al., 2021; Phuong et al., 2013).

Increasing dietary forage is also known to increase methane (CH_4_) yield (CH_4_/DMI) and intensity (CH_4_/milk yield) (Aguerre et al., 2011). Enteric fermentation from ruminant animals contributes approximately 40% towards global emissions from livestock supply chains (DEFRA, 2021; FAO, 2013). Whilst it is not possible to eradicate enteric CH_4_ emissions completely (Knapp et al., 2014), there is scope to reduce CH_4_ yield and intensity (Liu et al., 2017). Methane loss via microbial fermentation, represents an energy loss of 2% to 12% gross energy intake (GEI) (Johnson and Ward, 1996), thus emission mitigation would not only reduce environmental impact, but also enhance productivity and efficiency (Huws et al., 2018). Feeding concentrates has been a strategy to mitigate CH_4_ emissions by means of reducing CH_4_ yield and intensity. Aguerre et al. (2011) found that CH_4_ per kg of DMI was the highest in diets containing 68% compared to 53%, 63% and 47% FP (dry matter [DM] basis). Similarly, Olijhoek et al. (2018) observed higher CH_4_ per kg of DMI and energy corrected milk (ECM) in diets containing 68% compared to 39% forage (DM basis). Therefore, when investigating optimal dietary FP, it is important to consider the impact on CH_4_ production parameters.

Forage feeding can be an appropriate strategy to improve sustainability within the dairy system, by means of reducing the reliance on concentrate feeding, subsequently reducing feed costs, food-feed competition (when replacing human-edible concentrate feeds in the ration) and environmental impacts associated with the production and transportation of concentrate ingredients (Bijttebier et al., 2017). However, studies have shown that it may reduce productivity and increase CH_4_ yield and intensity (Aguerre et al., 2011; Olijhoek et al., 2018). Studies that have investigated the impact on feed efficiency are typically within the same experimental conditions (e.g. animal experiments) rather than a compilation of several datasets that would capture a wide range of FP ratios. As a result, studies focus on a relatively small FP range which may not account for both low-input and high intensity production systems (50% to 75% FP of DM by Broderick (2003), 20% to 50% FP DM by Lechartier and Peyraud (2010) or 51% to 90% FP DM by Olijhoek et al. (2022)). Additionally, the effects of varying FP are not observed for feed efficiency and CH_4_ production parameters under the same study.

It is also important to consider the impact of breed as they often differ in feed efficiency and CH_4_ production parameters (Olijhoek et al., 2022). This is particularly important because, in practice, production systems implementing high-forage diets are likely to utilise alternative genetic resources (e.g. using lower input breeds than Holstein-Friesian [HF]) (Butler, 2014). Previous investigations into the impact of FP and breed on CH_4_ emissions have only focused on the HF or Jersey breeds (Olijhoek et al., 2022, 2018). Thus, there is a requirement to identify an optimum forage feeding level which considers production intensity and animal factors such as breed. The current study aims 1) to investigate the impact of FP and animal breeding (including pure bred and crossbred cows) on productivity (milk yield, ECM), feed efficiency, and CH_4_ emission parameters, using data across a wide range of FP ratios and cows’ genetics and linear mixed effects models, 2) assess the relative impact of diet chemical composition and cow breed on productivity, feed efficiency, and CH_4_ emission parameters, using multivariate redundancy analysis, and 3) identify potential FP levels for optimum performance and reduced CH_4_ emissions, using linear and quadratic regression models. The study hypothesises that FP and breed will have an impact on feed efficiency and CH_4_ production parameters.

## 2 Materials and methods

### 2.1 Animals and diet

The current study utilized data from 32 individual experiments conducted at the Agri-Food and Biosciences Institute between 1992 and 2010 (references are listed in Appendix 1). The data set includes data from metabolism studies involving 796 HF cows (HF), 50 Norwegian Red cows (NR), 46 Jersey × HF crossbred cows (NR × HF) and 16 NR × HF crossbred cows (NR × HF), across 116 treatments. All crossbred animals used in the current study were the offspring from a previous crossbreeding program at the Agrifood and Biosciences Institute (Yan et al., 2006). Cows had a days in milk (DIM) average of 171 days (HF 177 days, J × HF 180 days; NR × HF 254 days, NR 166 days) and were of varying parities with 256 in first parity, 204 in second parity and 302 in parities 3 to 9.

Diets were offered ad libitum and consisted of either forage only (*n* = 65) or varying proportions of forage and concentrate (*n* = 843) depending on the experimental protocols of each experiment. Forages offered consisted of mostly grass silage (*n* = 583) and grass silage and maize silage (*n* = 154) with the remainder being; dried grass, straw, dried lucerne, fresh grass, whole crop wheat silage, maize silage, or dried grass/straw (*n* = 171). Grass silages, fresh and dried grass were produced from perennial ryegrass containing different varieties (Aberstar, Aberzest, Fetione, Magella, Menna, Merbo, Merlinda, and Spelga). Grass silages were either wilted or unwilted and ensiled with or without application of silage additives and were produced from primary growth, first regrowth and secondary regrowth material. Concentrates offered included a mineral-vitamin supplement along with cereal grains (barley, wheat, or maize), by-products (maize gluten meal, sugar-beet pulp with or without molasses, citrus pulp, or molasses), and protein feeds (soybean meal or rapeseed meal). The mean, standard variation and range of values for each animal and diet parameter used in the present study is shown in Table 1.

**Table 1:** Animal and diet data used in the present study.

Item	Mean^1^ (*n* = 908)	SD	Minimum	Maximum
**Animal data**				
BW, kg	553	66.9	379	757
BCS	2.53	0.385	1.50	4.50
DIM, d	171	92.8	18.0	554
Lactation number	2.42	1.475	1.00	9.00
**Feed intake and chemical content**				
DMI, kg/d	16.9	3.40	6.54	26.6
GEI, MJ/d	311	62.9	122	485
GE, MJ/kg of DM	18.4	0.59	15.9	21.3
ME, MJ/kg of DM	11.8	0.94	7.95	14.0
Forage DMI, kg/d	8.72	3.040	1.23	20.8
Concentrate DMI, kg/d	8.18	4.120	0	20.2
Forage proportion, kg/kg of DM	0.53	0.205	0.10	1.00
CP, kg/kg of DM	0.18	0.03	0.11	0.26
ADF, kg/kg of DM	0.23	0.045	0.11	0.36
NDF, kg/kg of DM	0.41	0.074	0.20	0.64
**Milk production and feeding efficiency**				
Milk yield, kg/d	22.4	7.89	1.00	49.1
ECM yield, kg/d	22.7	7.51	0.90	45.6
RFI, kg/d	−0.39	1.253	−5.19	3.67
Milk yield/DMI, kg/kg	1.31	0.345	0.15	2.75
ECM/DMI, kg/kg	1.33	0.332	0.14	3.34
Milk energy/DMI, MJ/kg	4.13	1.027	0.43	10.4
Milk yield/FDMI, kg/kg	2.97	1.671	0.26	18.6
Milk yield/CDMI, kg/kg	2.93	1.226	0.19	8.38
**Methane emissions**				
CH_4_, g/d	370	78.8	136	672
CH_4_/DMI, g/kg	22.3	4.14	8.21	43.7
CH_4_/MY, g/kg	18.7	9.36	5.83	198
CH_4_/ECM, g/kg	18.1	9.44	5.56	219
CH_4_ energy/GEI, MJ/MJ	0.07	0.013	0.03	0.14

BW = body weight; BCS = body condition score; DIM = days in milk; DMI = dry matter intake; GEI = gross energy intake; GE = gross energy; DM = dry matter; ME = metabolizable energy; CP = crude protein; ADF = acid detergent fibre; NDF = neutral detergent fibre; ECM = energy corrected milk; RFI = residual feed intake; FDMI = forage DMI; CDMI = concentrate DMI; CH_4_ = methane; MY = milk yield; SD = standard deviation.

1 Means were calculated from 908 observations. Means for ADF, NDF, and CP (kg/kg DM) were calculated from 898, 879 and 872 observations, respectively. Means for milk yield/CDMI were calculated from 843 observations.

### 2.2 Digestibility study, calorimetric measurements and sample analysis

All experiments used indirect open-circuit respiration calorimeter chambers to measure energy intake and output of individual cows. Cows were housed as a group in cubicle accommodation with free access to water and were offered their experimental diets for at least 3 weeks, to ensure acclimatisation to diets, before being taken to individual digestibility stalls. For digestibility measurements, animals were transferred to a tie-stall facility for 5 to 8 days where measurements were taken during the final 3 to 6 days for feed intake, and total faeces and urine output for each individual animal. Faeces and urine were measured, recorded and sampled daily (15% of total excretion). Sulfuric acid was added (35% H_2_SO_4_) to the urine containers to prevent loss of nitrogen (N) as ammonia (10 to 35 mL/container to maintain the pH value of urine <3). After the digestibility periods, animals were transferred to individual calorimeter chambers for 3 to 5 days for measurements for gaseous exchange measurements (CH_4_, CO_2_, and O_2_) during the final 2 to 4 days. All equipment, sampling procedures, analytical methods, and calculations used in the calorimetric studies were described by Gordon et al. (1995) and calibration of the chambers by Yan et al. (2000).

### 2.3 Calculations and statistical analysis

A number of feed efficiency parameters were calculated in the present study. Energy corrected milk yield was calculated based on the following equation:

ECM(kg/d)=actualmilkenergyoutput(MJ/d)/standardmilkenergycontent(MJ/kg)

where

$$\text{Actual}\;\text{milk}\;\text{energy}\;\text{output}\;\left(\text{MJ}/d\right)=\text{milk}\;\text{yield(kg/d)}\times \text{milk}\;\text{energy}\;\text{content (MJ/kg)}\;\text{measured}\;\text{by}\;\text{isoperibol}\;\text{bomb}\;\text{calorimeters.}$$

The standard milk energy content was calculated based on the assumption of 1 kg of standard milk from Holstein cows, containing 40 g fat, 32 g crude protein (CP) and 48 g lactose, and the equation of Tyrrell and Reid (1965):

Standardmilkenergycontent(MJ/kg)=(40×0.0384)+(32×0.0223)+(48×0.0199)–0.108.

Residual feed intake (RFI) was calculated from the following equation:

RFI(kg/d)=actualDMI(kg/d)–predictedDMrequirement(kg/d)

where predicted DM requirement was calculated from total metabolizable energy (ME) requirements for maintenance, lactation, body weight (BW) change and pregnancy, predicted using models of Feed into Milk (FiM) (Thomas, 2004) divided by actual dietary ME concentration measured in the present study.

PredictedDMrequirement(kg/d)=predictedtotalMErequirement(MJ/d)/actualdietMEcontent(MJ/kgDM).

All efficiency measures were calculated using actual intake as follows:

Milkyield/forageDMI(FDMI,kg/kg)=milkyield(kg/d)/DMIfromforage(kg/d);

Milkyield/concentrateDMI(CDMI,kg/kg)=milkyield(kg/d)/DMIfromconcentrates(kg/d).

**Fig. 1:**
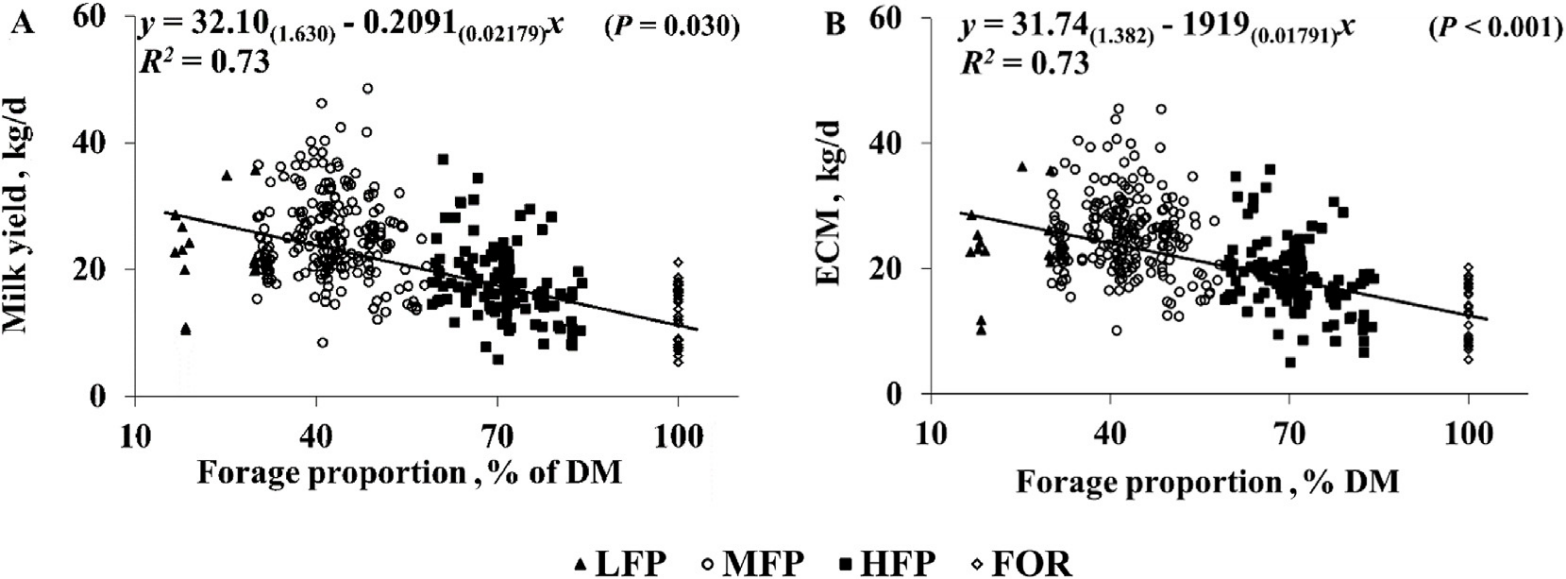
Relationship between forage proportion and (A) milk yield and (B) ECM for individual cows offered four experimental groups of forage proportion. LFP = 10% to 30% FP of DMI; MFP = 30% to 59% FP of DMI; HFP = 60% to 87% FP of DMI; FOR = 100% FP of DMI. DM = dry matter; ECM = energy corrected milk yield; DMI = dry matter intake.

**Fig. 2:**
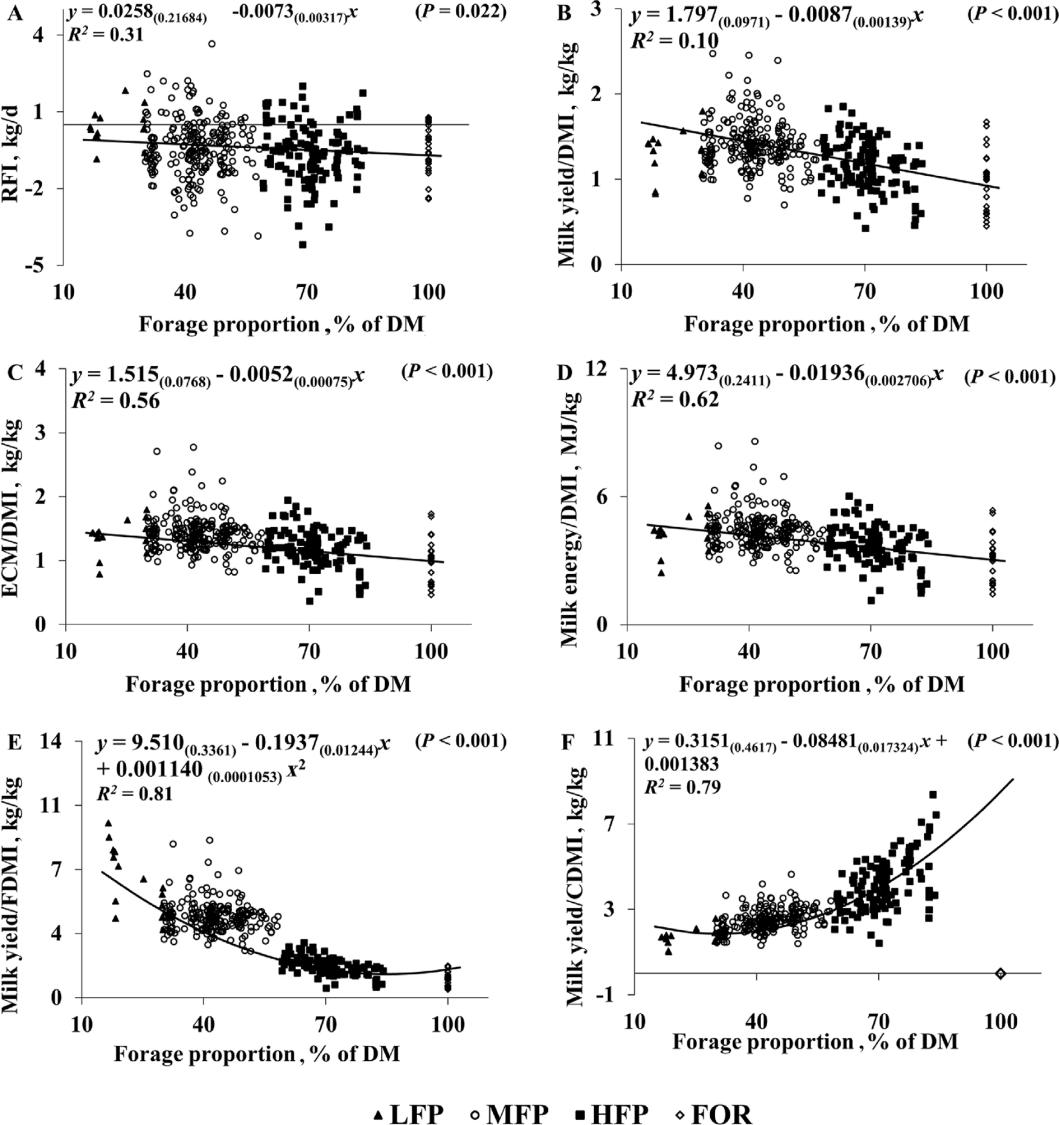
Relationship between forage proportion and (A) RFI, (B) milk/DMI, (C) ECM/DMI, (D) milk energy/DMI, (E) milk/forage DMI and (F) milk/concentrate DMI for individual cows offered four experimental groups of forage proportion. LFP = 10% to 30% FP of DMI; MFP = 30% to 59% FP of DMI; HFP = 60% to 87% FP of DMI; FOR = 100% FP of DMI. RFI = residual feed intake; DM = dry matter; DMI = DM intake; ECM = energy corrected milk yield; FDMI = forage DMI; CDMI = concentrate DMI.

**Fig. 3:**
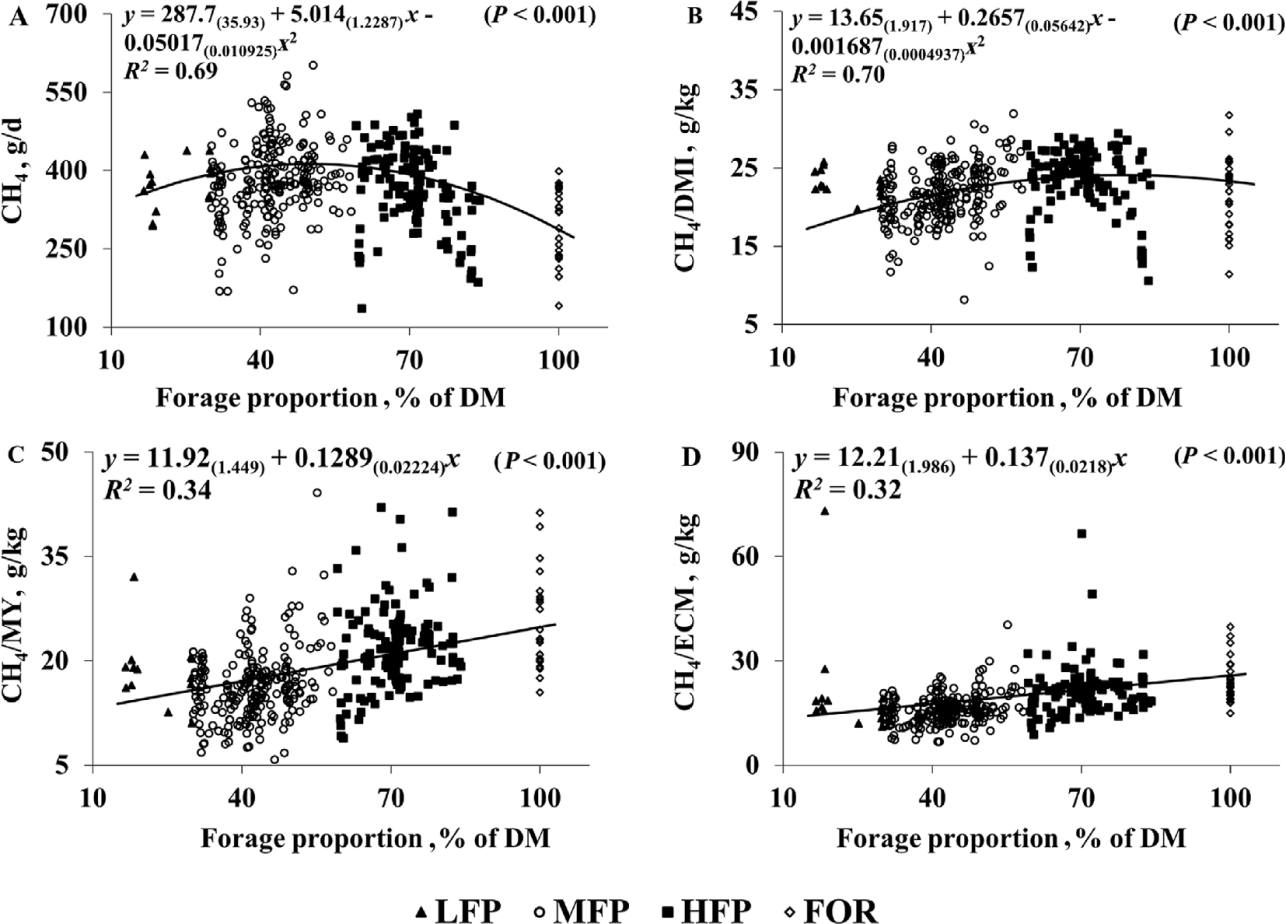
Relationship between forage proportion and (A) CH_4_, (B) CH_4_/DMI, (C) CH_4_/milk yield, (D) CH_4_/ECM for individual cows offered four experimental groups of forage proportion. LFP = 10% to 30% FP of DMI; MFP = 30% to 59% FP of DMI; HFP = 60% to 87% FP of DMI; FOR = 100% FP of DMI. CH_4_ = methane; DMI = dry matter intake; DM = dry matter; MY = milk yield; ECM = energy corrected milk yield.

**Fig. 4:**
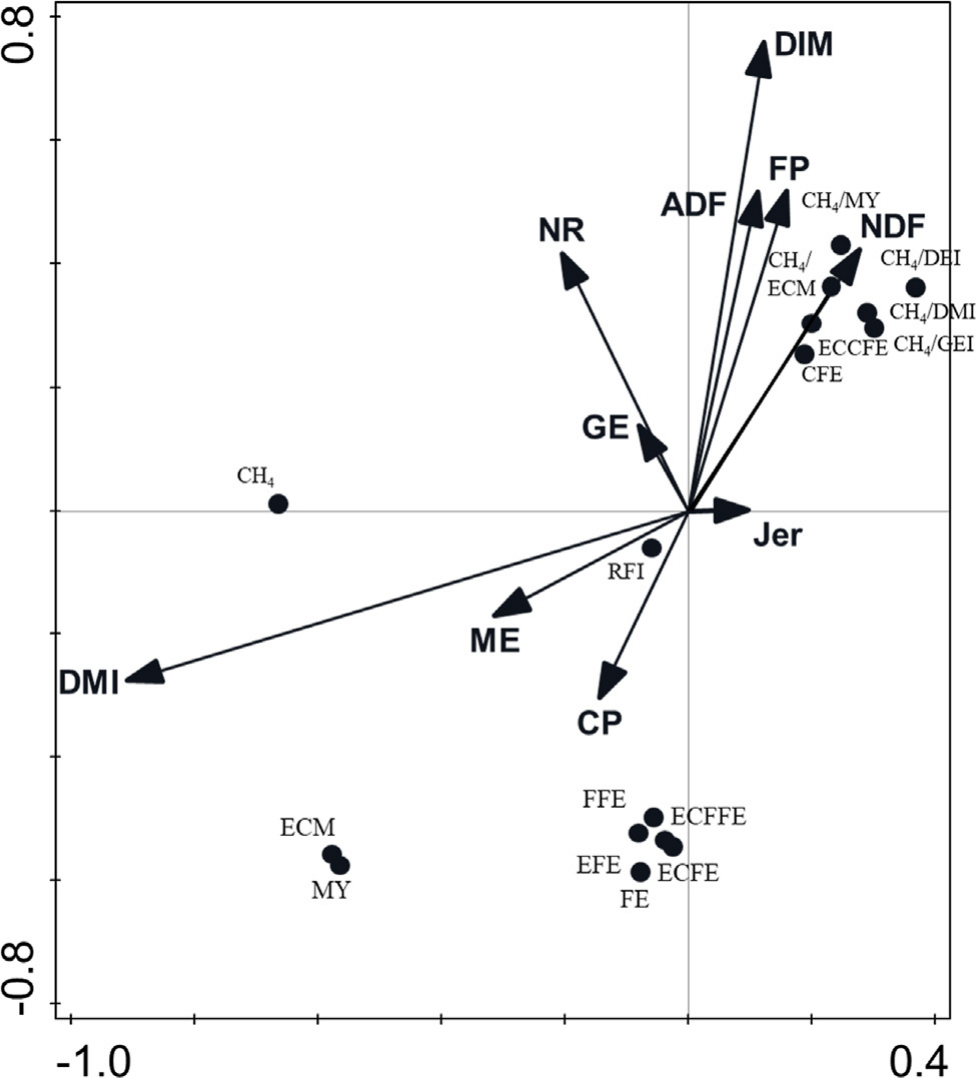
Biplot derived from the redundancy analysis, showing the relationship between diet composition parameters (DMI, GE, ME, NDF, ADF, CP and FP), animal factors (DIM), breed (JER, NR), relative to productivity parameters (MY, ECM), feed efficiency parameters (RFI, FE, CFE, FFE, ECFE, ECFFE, ECCFE, EFE) and methane production parameters (CH_4_, CH_4_/DMI, CH_4_/milk yield, CH_4_/ECM, CH_4_ energy/DEI, CH_4_ energy/GEI). The total adjusted explained variation was 45.2%. Axis 1 explained 44.4% of the variation and axis 2 explained a further 1.34% of the variation. Continuous variables, shown as arrows were (presented in order of contribution to the explained variation and *P*-value in parentheses): DMI (36.8%, *P* = 0.002), NR (3.52%, *P* = 0.002), ME (1.35%, *P* = 0.002), CP (1.50%, *P* = 0.002), DIM (1.19%, *P* = 0.002), ADF (0.93%, *P* = 0.002), JER (0.21%, *P* = 0.072), FP (0.17%, *P* = 0.144), NDF (0.18%, 0.088), GE (0.05%, *P* = 0.420). DMI = dry matter intake; GE = gross energy; ME = metabolizable energy; NDF = neutral detergent fibre; ADF = acid detergent fibre; CP = crude protein; FP = forage proportion; DIM = days in milk; JER = Jersey; NR = Norwegian Red; MY = milk yield; ECM = energy corrected milk yield; RFI = residual feed intake; FE = milk yield/DMI; CFE = milk yield/concentrate DMI; FFE = milk yield/forage DMI; ECFE = ECM/DMI; ECFFE = ECM/forage DMI; ECCFE = ECM/concentrate DMI; EFE = milk energy/DMI; CH_4_ = methane production; DEI = digestible energy intake; GEI = gross energy intake.

All data points were included in the analysis (*n* = 908). A linear mixed model (residual maximum likelihood analysis [REML]) was used to investigate the effect of FP, and breed on productivity, feed efficiency, and CH_4_ production parameters. Data were separated into 4 groups based on FP (DM basis); low (LFP; 10% to 30%, *n* = 40), medium (MFP; 30% to 59%, *n* = 551), high (HFP; 60% to 87%, *n* = 243) and pure (FOR; 100%, *n* = 65) FP. The statistical programme used was GenStat 23 (VSN International, 2020). The fixed effects were FP (LFP, MFP, HFP and FOR) and breed (HF, J × HF, NR, NR × HF), while experiment and cow (nested in experiment) were included as the random effects. Normality of the residuals for all variables was assessed visually using a normality plot and histogram of residuals, and all variables were found to be normally distributed and were statistically analysed as untransformed values. When the fixed effect was significant for a measured variable (*P* < 0.05), pairwise comparisons of means were performed using Fisher's LSD test (*P* < 0.05). Means for animal, diet composition and intake for each treatment group for FP and breed are outlined in Table 2. Regression equations were developed using REML so that the potential random effects for cow, experiment, forage proportion, breed, forage type and parity could be accounted for. The response variables were productivity, feed efficiency and CH_4_ production parameters that were significantly affected by dietary FP in the REML analysis; while FP (expressed as %, DM basis) was the explanatory variable (Fig. 1, Fig. 2, Fig. 3). Both linear and quadratic regressions were tested. If the quadratic effect was statistically significant, the quadratic relationship is presented; or else the linear relationship is presented. Multivariate redundancy analysis (RDA) was carried out using (Canoco5, 2012) to further investigate the impact of animal parameters and dietary drivers on productivity, feed efficiency and CH_4_ production parameters. The arrow lengths and directions represent the correlations between the driver variables (dietary composition and animal parameters including breed and DIM) and response variables (productivity, feed efficiency and CH_4_ production parameters) (Fig. 4).

**Table 2:** Effect of forage proportion and breed on animal, diet and feed intake parameters.

Item	FP^1^						Breed^2^					
	LFP	MFP	HFP	FOR	SE	*P*-value	HF	J × HF	NR × HF	NR	SE	*P*-value
	(*n* = 49^3^)	(*n* = 551^3^)	(*n* = 243^3^)	(*n* = 65^3^)			(*n* = 796^3^)	(*n* = 46^3^)	(*n* = 16^3^)	(*n* = 50^3^)		
**Animal data**												
Holstein-Friesian	100	88.4	80.2	100			100	0	0	0		
Jersey × Holstein-Friesian	0	4.72	8.23	0			0	100	0	0		
Norwegian Red × Holstein-Friesian	0	1.45	3.29	0			0	0	100	0		
Norwegian Red	0	5.44	8.23	0			0	0	0	100		
BW, kg	573	561	564	542	17.7	0.565	559^b^	518^c^	615^a^	548^bc^	20.6	0.017
BCS	2.80^ab^	2.81^a^	2.69^b^	2.72^ab^	0.126	<0.001	2.52^b^	2.70^ab^	2.93^a^	2.87^a^	0.128	0.001
DIM	137	147	174	212	23.4	0.003	177^b^	180^b^	254^a^	166^b^	20.2	0.009
**Diet chemical composition**												
GE, MJ/kg	18.5	18.4	18.5	18.8	0.20	0.317	18.5	18.5	18.7	18.5	0.07	0.101
ME, MJ/kg	12.1	11.9	11.6	11.0	0.33	<0.001	11.6	11.6	11.9	11.5	0.13	0.302
CP, kg/kg of DM	0.20^a^	0.19^a^	0.17^b^	0.16^b^	0.008	<0.001	0.18	0.18	0.18	0.18	0.004	0.952
ADF, kg/kg of DM	0.21^c^	0.21^c^	0.27^b^	0.31^a^	0.011	<0.001	0.25	0.25	0.26	0.25	0.004	0.570
NDF, kg/kg of DM	0.36^c^	0.37^c^	0.46^b^	0.54^a^	0.020	<0.001	0.43	0.43	0.44	0.43	0.005	0.439
**Feed intake**												
Forage proportion, % DMI	36.4^d^	42.3^c^	68.9^b^	100.0^a^	0.30	<0.001	61.9	61.9	62.0	62.0	1.60	0.100
CP intake, kg/d	3.42^a^	3.40^a^	2.66^b^	2.36^b^	0.275	<0.001	2.91	3.08	3.07	2.79	0.172	0.441
ADF intake, kg/d	3.65	3.82	4.09	4.22	0.011	<0.001	3.89	3.98	4.18	3.73	0.004	0.291
NDF intake, kg/d	6.29^b^	6.50^b^	7.09^a^	7.51^b^	0.020	<0.001	6.77	7.15	7.09	6.38	0.311	0.153

FP = forage proportion; BW = body weight; BCS = body condition score; DIM = days in milk; DM = dry matter; GE = gross energy; ME = metabolizable energy; CP = crude protein; ADF = acid detergent fibre; NDF = neutral detergent fibre; DMI = dry matter intake; SE = standard error.

^a-^^d^ Means within a row and fixed factor with different superscript letters are significantly different according to Fisher’s protected least significant difference test (*P* < 0.05).

1 LFP = 10% to 30% FP of DMI; MFP = 30% to 59% FP of DMI; HFP = 60% to 87% FP of DMI; FOR = 100% FP of DMI.

2 HF = Holstein-Friesian; J × HF = Jersey × Holstein- Friesian; NR × HF = Norwegian Red × Holstein Friesian; NR = Norwegian Red.

3 *n* is the number of records used to calculate means ± SE and *P*-values.

## 3 Results

### 3.1 Effect of forage proportion

#### 3.1.1 Animal parameters and feed intake

All feed intake parameters varied between treatments; total DMI and GEI was higher for cows consuming LFP and MFP compared to HFP (+2.0 and +2.6 kg/d for total DMI (*P* < 0.001) and +40 and +47 MJ/d for GEI (*P* < 0.001)) and FOR (+3.5 and +4.1 kg/d for total DMI and +63 and +70 MJ for GEI (*P* < 0.001)) (Table 3). Forage DMI increased significantly as FP increased from LFP to MFP (+1.25 kg/d), to HFP (+3.14 kg/d), to FOR (+3.10 kg/d) (*P* < 0.001) (Table 3). Concentrate DMI was higher for LFP and MFP compared to HFP (+6.78 and +5.66 kg/d, respectively) and FOR (+11.4 and +10.3 kg/d) and higher for HFP compared to FOR (+4.64 kg/d) (*P* < 0.001) (Table 3).

**Table 3:** Effect of dietary forage proportion and breed on feed intake, productivity, efficiency parameters and methane production in lactating dairy cows.

Item	FP^1^				SE	*P*-value	Breed^2^				SE	*P*-value
	LFP (*n* = 49^3^)	MFP (*n* = 551^3^)	HFP (*n* = 243^3^)	FOR (*n* = 65^3^)			HF (*n* = 796^3^)	J × HF (*n* = 46^3^)	NR × HF (*n* = 16^3^)	NR (*n* = 50^3^)		
**Feed intake**												
Total DMI, kg/d	17.3^a^	17.9^a^	15.3^b^	13.8^b^	1.16	<0.001	16.0	16.4	16.6	15.2	0.76	0.474
GEI, MJ/d	322^a^	329^a^	282^b^	259^b^	21.6	<0.001	296	304	311	282	14.0	0.400
Forage DMI, kg/d	6.31^d^	7.56^c^	10.7^b^	13.8^a^	0.76	<0.001	9.55	9.74	9.90	9.05	0.432	0.393
Concentrate DMI, kg/d	11.40^a^	10.31^a^	4.64^b^	0.00^c^	0.752	<0.001	6.54	6.81	6.78	6.28	0.466	0.762
**Milk production and feeding efficiency**												
Milk yield, kg/d	25.0^a^	22.6^a^	17.5^b^	11.9^b^	2.47	<0.001	20.7	18.8	19.0	18.4	1.61	0.174
ECM yield, kg/d	25.1^a^	23.6^a^	18.3^b^	12.7^c^	2.32	<0.001	20.8	21.2	19.1	18.5	1.56	0.290
RFI, kg/d	0.56	−0.18	−0.42	−0.50	0.410	0.020	−0.26^b^	−0.28^b^	0.50^a^	−0.51^b^	0.249	0.040
Milk yield/DMI, kg/kg	1.40^a^	1.26^a^	1.15^b^	0.89^b^	0.116	<0.001	1.27	1.14	1.12	1.18	0.072	0.041
ECM/DMI, kg/kg	1.43^a^	1.32^a^	1.20^b^	0.95^b^	0.112	<0.001	1.28	1.28	1.14	1.20	0.068	0.238
Milk energy/DMI, MJ/kg	4.43^a^	4.10^a^	3.72^b^	2.93^b^	0.256	<0.001	3.97	3.97	3.53	3.72	0.228	0.238
Milk yield/FDMI, kg/kg	5.33^a^	3.21^b^	1.72^c^	0.77^d^	0.371	<0.001	2.97	2.65	2.64	2.77	0.248	0.288
Milk yield/CDMI, kg/kg	2.05^b^	2.09^b^	4.09^a^	—4	0.241	<0.001	2.95	2.67	2.61	2.76	0.176	0.096
**Methane emissions**												
CH_4_, g/d	384^ab^	397^a^	371^b^	316^b^	27.9	<0.001	348	376	385	360	18.5	0.129
CH_4_/DMI, g/kg	22.5^ab^	22.4^b^	24.3^a^	22.9^ab^	1.47	<0.001	22.1^b^	23.0^ab^	23.2^ab^	23.9^a^	0.87	0.048
CH_4_/MY, g/kg	21.0^ab^	19.1^b^	22.5^a^	27.0^a^	2.72	<0.001	20.3	22.5	23.3	23.5	1.93	0.085
CH_4_/ECM, g/kg	21.1^ab^	17.9^b^	21.6^a^	25.8^a^	2.53	<0.001	20.0	20.4	22.8	23.3	1.99	0.176
CH_4_ energy/GEI, MJ/MJ	0.068	0.068	0.074	0.069	0.0044	<0.001	0.067	0.070	0.069	0.072	0.0026	0.056

FP = forage proportion; GEI = GE, intake; DMI = dry matter intake; ECM = energy corrected milk; RFI = residual feed intake; FDMI = forage DMI; CDMI = concentrate DMI; CH_4_ = methane; MY = milk yield; SE = standard error.

^a-d^ Means within a row and fixed factor with different superscript letters are significantly different according to Fisher’s protected least significant difference test (*P* < 0.05).

1 LFP = 10% to 30% FP of DMI; MFP = 30% to 59% FP of DMI; HFP = 60% to 87% FP of DMI; FOR = 100% FP of DMI.

2 HF = Holstein-Friesian; J × HF = Jersey × Holstein- Friesian; NR × HF = Norwegian Red × Holstein- Friesian; NR = Norwegian Red.

3 *n* is the number of records used to calculate means ± SE and *P*-values.

4 No value for milk yield/CDMI in FOR treatment due to diet being 100% forage.

#### 3.1.2 Productivity and feed efficiency

Milk yield, milk yield/DMI, ECM/DMI and milk energy/DMI were higher (*P* < 0.001) for LFP and MFP compared to HFP (+7.5 and +5.1 kg/d for milk yield, +0.25 and +0.11 kg/kg for milk yield/DMI and +0.23 and +0.12 kg/kg for ECM/DMI and +0.72 and +0.38 MJ/kg for milk energy/DMI) and FOR, treatments (+0.51 and +0.37 kg/d for milk yield, +0.48 and +0.37 kg/kg for milk yield/DMI and +0.23 and +0.12 kg/kg for ECM/DMI and +1.50 and +1.17 MJ/kg for milk energy/DMI). Treatments LFP and MFP had higher ECM (*P* < 0.001) compared to HFP (+6.8 and +5.3 kg) and FOR (+12.4 and +2.40 kg). Forage efficiency (milk yield/FDMI) decreased (*P* < 0.001) from LFP to MFP (−2.12 kg/kg) to HFP (−1.49 kg/kg) to FOR (−0.95 kg/kg). Feed concentrate efficiency (milk yield/concentrate DMI) was higher (*P* < 0.001) for the HFP compared to LFP (+2.04 kg/kg) and MFP (+2.00 kg/kg). Residual feed intake was significantly affected (*P* = 0.020) by treatments but pairwise comparison did not identify any differences between groups. Correlations between FP and productivity parameters are shown in Fig. 1. Correlations between FP and efficiency parameters are shown in Fig. 2

#### 3.1.3 Methane production

Methane production was higher (*P* < 0.001) for MFP compared to HFP and FOR (+26 and +81 g/d) but there was no difference between LFP and MFP or between LFP, HFP and FOR. Methane yield (CH_4_/DMI) was higher (*P* < 0.001) for HFP compared to MFP (+1.9 g/kg). However, there were no differences between HFP, FOR and LFP, or between MFP and LFP and FOR. Methane intensity (CH_4_/milk yield and CH_4_/ECM) was highest (*P* < 0.001) for treatments HFP and FOR compared to MFP (+3.4 and +7.9 g/kg for CH_4_/milk yield and +3.7 and +7.9 g/kg for CH_4_/ECM). However, LFP did not result in differences in CH_4_ intensity compared with MFP or with HFP and FOR. Correlations between FP and CH_4_ production parameters are shown in Fig. 3.

### 3.2 Effect of breed

Norwegian Red × HF cows had the highest RFI (*P* = 0.040) compared to HF (+0.76 kg/d), J × HF (+0.78 kg/d) and NR (+1.01 kg/d). Breed had a significant effect on milk yield/DMI (*P* = 0.041) but pairwise comparisons detected no differences between breeds. Norwegian Red cows had higher CH_4_/DMI (*P* = 0.048) compared to HF cows (+1.8 g/kg) but both breeds had similar values to J × HF and NR × HF breeds.

### 3.3 Multivariate analysis of the effect of animal and dietary drivers on productivity, efficiency and methane production parameters

The RDA biplot showing the relative impact of animal and dietary drivers on productivity, efficiency and CH_4_ production parameters is demonstrated in Fig. 4. Drivers together explained 45.2% of the total adjusted variation, of which 44.4% was explained by axis 1 and a further 1.34% was explained by axis 2. Dry matter intake accounted for 36.8% of the variation whilst NR, dietary ME, CP and DIM accounted for 3.52%, 1.35%, 1.50% and 1.19%, respectively. The other individual drivers explained <1% of the variation each. Dry matter intake and ME was negatively correlated with CH_4_ production parameters; CH_4_/DMI, CH_4_/milk yield, CH_4_/ECM, CH_4_-E/GEI, CH_4_-E/DEI and concentrate feed efficiency. The same response variables were positively correlated with dietary drivers; ADF, NDF and FP as well as animal drivers; DIM and NR genetics. These drivers were also negatively correlated with productivity responses milk yield, ECM and CH_4_ production. Feed efficiency responses (milk yield/DMI, milk yield/FDMI, ECM/DMI, ECM/FDMI, milk energy/DMI) were positively correlated with dietary CP content and ME and negatively correlated with dietary ADF, NDF and FP as well as animal drivers; DIM and to a lesser extent NR genetics.

## 4 Discussion

### 4.1 Effect of forage proportion

#### 4.1.1 Feed intake

Due to the bulk density and increased rumen fill associated with forage NDF of the diet, feed intake is often reduced as FP increases (Allen, 2000; Li et al., 2020; Yang and Beauchemin, 2007a). Results from the current study support this; DMI reduced when FP increased from 10% to 30% and 30% to 59% to 60% to 87% and 100% of DMI. Despite similar results for DMI between the LFP and MFP groups and the HFP and FOR groups, no quadratic relationship was found and regression identified linear reductions in DMI as FP increased. However, a more dramatic decrease in DMI is seen when FP is increased from between 30% and 59% to between 60% and 87%. Reductions in DMI are primarily as a result of increased rumen fill associated with increased intake of NDF from forage diets (Allen, 2000). Previous investigations have found DMI reduction when FP increased from 35% to 60% of DM (Li et al., 2020; Yang and Beauchemin, 2007a). However, some investigations have found similar DMI between diets of differing FP; Olijhoek et al. (2022) found no differences in DMI as concentrate proportion increased from 49% to 70% to 91% of DM and Lechartier and Peyraud (2010) found no further increases in DMI when forage proportion was reduced from 35% to 20% of DM. However, the current study found similar DMI between treatments LFP and MFP and between treatments HFP and FOR. Similar results for feed intake between the LFP and MFP in the current study, along with observed reductions in rate of increase in feed intake, were observed by Olijhoek et al. (2022) when FP was reduced below 51%, and by Lechartier and Peyraud (2010) when FP was reduced below 35%. These findings could be the result of animals voluntarily reducing their intake in response to increases in ruminal propionate or a lower pH in diets containing more concentrate and less forage (Lechartier and Peyraud, 2010). Lechartier and Peyraud (2010) also investigated the impact of diets with lower fibre content than the recommended by NRC (2001) (minimum of 25% NDF in DM); in the case of Lechartier and Peyraud (2010), diets containing a FP <20% of DM, had 7.6% NDF in DM resulting in observed ruminal pH values below 5.8 along with milk fat depression. Lechartier and Peyraud (2010) suggested that these findings may have indicated sub-acute ruminal acidosis (SARA) and may have contributed to the lack of increase in DMI as FP reduced below 35% of DM. Considering that the minimum NDF proportion in the current study was below 25% of DM, it is possible that SARA may have occurred in cows consuming the LFP treatment, contributing to lack of increase in DMI or milk yield. However, the current study did not measure ruminal pH, milk fat or investigate ruminal proportions of volatile fatty acid (VFA) and thus it would not be possible to confirm whether any cows had SARA. It is also possible that particle length and physically effective NDF may have influenced DMI (Lechartier and Peyraud, 2010). These have not been accounted for in the current study, and are likely to have contributed to the effects of FP observed here (Krause et al., 2002; Lechartier and Peyraud, 2010; Yang and Beauchemin, 2007a).

#### 4.1.2 Productivity and feed efficiency

Investigations into the impact of FP on productivity have been relatively consistent, finding reduced milk production as forage proportion increases (Lechartier and Peyraud, 2010; Yang and Beauchemin, 2007b). Increases in milk yield are primarily a result of the higher DMI of lower forage diets. Results from multivariate analysis corroborate findings here showing negative correlations between FP and productivity measures (milk yield and ECM). Similarly, regression analysis showed linear reductions in milk yield with increasing FP, with milk yield decreasing by 0.21 kg/d with each 1% increase in FP (Fig. 1). However, the current study did not report any differences in milk yield between LFP and MFP or between HFP and FOR, probably due to the similar DMI and subsequent similar intakes of chemical constituents (CP, ADF and NDF) between these same treatments. Results here are consistent with previous investigations observing increases in NDF and ADF intake as FP increases (Olijhoek et al., 2022; Yang and Beauchemin, 2007b). Results here confirm previous investigations suggesting that whilst feeding high proportions of concentrate (30% to 59% of DMI in the current study) may result in improvements in productivity (higher milk and ECM yields) (Yang and Beauchemin, 2007b), any further increase would not result in any significant benefit. Olijhoek et al. (2022) found no differences in feed intake and subsequently milk yield as concentrate feeding level increased from 49% to 70% to 91% of DM; but the high-concentrate diets were probably excessive to what is typically fed to dairy cattle. The current study investigated a much wider range (FP range 10% to 100% of DMI) finding no further improvements in milk yield by reducing FP below 30% to 59% of DMI or amending FP within the range of 60% to100% (DM basis). Considering the expense of outsourcing concentrates (Hayes et al., 2013), feeding a diet consisting of between 10% and 30% forage in DM would not offer any benefits to profitability compared to a diet of 30% to 59% forage in DM.

It is also important to consider the impacts of dietary CP that is often supplied through supplemental concentrate feeding, and is known to increase milk yield (Broderick, 2003). Therefore, it is not surprising that treatments in the current study with lower concentrate feeding level would have lower CP (% of DM) and thus lower intakes of CP in higher FP treatments as also observed in previous studies (Lechartier and Peyraud, 2010). Additionally, the impact of dietary starch is well documented, with studies reporting increased productivity with higher starch proportions (Yang and Beauchemin, 2006), and may differ between forage and concentrate type (Kliem et al., 2008; Sterk et al., 2011). Since the current study utilized data from 32 experiments varying in forage (grass silage, maize silage, dried grass, straw, dried lucerne, fresh grass, whole crop wheat silage, dried grass/straw), and concentrate type, it is likely that starch differences might have affected the observations. Whilst starch was not measured in the current study, higher dietary NDF is often coupled with decreases in starch content, along with reductions in ME (Beckman and Weiss, 2005). Redundancy analysis results showed positive associations between productivity parameters (milk yield and ECM) and dietary CP content and ME and negative correlations with dietary ADF, NDF content and FP. Therefore, it is possible that similarities between intakes of CP (3.42 and 3.40 kg/d) alongside NDF (6.29 and 6.50 kg/d) and ADF (3.65 and 3.82 kg/d) intakes between treatments LFP and MFP could have contributed to the similar milk yields observed between these treatments. As with most higher forage diets, ME was numerically lower in the higher FP, as a result of concentrate feeds being more energy dense, with the LFP diet containing 1.1 MJ more per kg of DM than the FOR treatment. This, alongside other dietary chemical components (CP, NDF and ADF) would have influenced productivity and efficiency variables (Dong et al., 2015).

Whilst it is well known that increasing FP in the diet reduces milk and ECM yield (Lechartier and Peyraud, 2010; Olijhoek et al., 2022) as also observed in the current study, findings relating to feed efficiency are somewhat inconsistent. Many investigations have found that cows produced more milk yield/DMI as FP or NDF is reduced (Lechartier and Peyraud, 2010) in agreement with the current study finding that cows produced more milk yield/DMI when FP was between 10% to 30% and 30% to 59% of DMI compared to 60% to 87% and 100% of DMI. Additionally, regression analysis suggests that increasing FP by 10% would reduce milk yield/DMI by 0.09 kg/d. Results from RDA also demonstrate that increases in FP and subsequent increased content of ADF and NDF components is negatively correlated with efficiency parameters (with the exception of concentrate feed efficiency [milk/kg concentrate DMI]). However others have found no effect on milk yield/DMI when comparing 80% to 35% (Moorby et al., 2006) and 60% to 35% FP (of DM) (Li et al., 2020). Although, in the case of Li et al. (2020), ECM/DMI was higher when FP increased from 35% to 60% (of DM), but probably because of reduced DMI coupled with higher milk fat content from cows consuming higher forage diets. The current study reports the opposite, with cows consuming 10% to 30% and 30% to 59% forage in DMI producing more milk, ECM and milk energy per kg of DMI compared to 60% to 87% and 100% forage in DMI. This can be explained by the concurrent reduction in DMI and GEI following increased rumen fill as a result of lower energy dense forage diets alongside the reduction in milk yield (Allen, 2000). Values for feed efficiency were similar between LFP and MFP groups, suggesting that concentrate feeding between the range of 90% to 70% of DM would not result in any further benefits to productivity or feed efficiency. Some investigations have suggested that the feed efficiency of low-efficiency cows (classified as animals with high RFI [voluntary intake is higher than nutritional requirements for maintenance and lactation]) may benefit by means of reducing DMI without a simultaneous reduction in milk yield (Ben Meir et al., 2021). Previous work has observed higher net energy captured in milk (+0.16 MJ) per kg of digestible energy intake when low efficiency cows (RFI >0.5) were offered a higher forage NDF diet (23.4% compared to 17.5% forage NDF [DM basis]) (Ben Meir et al., 2021).Whilst the current study did not investigate the impact of FP on low efficiency animals specifically, there was a significant difference in RFI between treatment groups. Residual feed intake reduced as FP increased, suggesting that animals on the higher forage diets consumed less than their requirements for maintenance and lactation. Results for feed efficiency are statistically similar between LFP and MFP groups, but numerically, cows in the LFP group consumed 0.56 kg DM more than their requirements compared to cows in the MFP group which consumed 0.18 kg less DM than their requirement but still maintained milk production. Additionally, similarities in feed efficiency between HFP and FOR suggest that cows in lower intensity systems consuming between 60% and 87% forage in DMI may not see reductions in feed efficiency if FP were to increase to close to 100% of DMI.

#### 4.1.3 Methane production parameters

Methane production often increases as DMI increases (Marumo et al., 2023). Previous investigations have found reductions in DMI in response to increasing FP due to increased bulk density of NDF (Aguerre et al., 2011). Subsequently CH_4_ production is often reduced as FP increases (Aguerre et al., 2011) in agreement with the current study; decreased CH_4_ production as FP increased from MFP to HFP. Whilst no differences were detected between LFP and MFP treatments, regression analysis suggested quadratic effects with CH_4_ reaching a maximum of 411 g/d when diet FP was 45% of DM. Aguerre et al. (2011) reported increased CH_4_ production as FP increased from 47% to 68% (of DM). Differences between Aguerre et al. (2011) and the current study could be attributed to the similar intakes of DMI between diets in Aguerre et al. (2011). This resulted in increased intakes of NDF as FP increased. Investigations have suggested that diets containing high concentrate proportions and thus starch, favour fermentation pathways which produce propionate and consume metabolic H_2_ subsequently reducing its availability for methanogenesis and reducing CH_4_ production (Knapp et al., 2014). Whereas high-forage diets and thus higher NDF diets, encourage fermentation pathways which produce acetate and butyrate as well as H_2_ which methanogens utilise, producing CH_4_ (Knapp et al., 2014). Although, based on results from RDA in the current study, it seems that DMI is a stronger driver than NDF for CH_4_ production. Interestingly, CH_4_ production did not differ between LFP, HFP and FOR in the current study despite DMI being significantly higher between LFP and the two higher FP groups (HFP and FOR). An explanation could be that whilst DMI did not reduce significantly, FP between 10% and 30% of DMI resulted in a numerical reduction in DMI of −0.9 kg/d and thus a reduction in NDF intake of −0.21 kg/d compared to a FP of 30% to 59%. Additionally, pathways which favour the production of propionate (high-concentrate diets and thereby starch) often result in a lower ruminal pH which can subsequently inhibit methanogens resulting in reduced CH_4_ production (Janssen, 2010) potentially explaining similarities in CH_4_ between LFP and the two high FP groups (HFP and FOR). It is also possible that diet digestibility might have influenced CH_4_ production since studies have associated increased CH_4_ production with increased digestibility (Olijhoek et al., 2018) and that digestibility was also heavily influenced by dietary fibre (Yang and Beauchemin, 2007a), which was indeed higher with increasing FP in the diet especially in the HFP and FOR groups.

The findings of lower CH_4_ yield and intensity as FP decreases are consistent with previous findings (Aguerre et al., 2011; Jiao et al., 2014; Olijhoek et al., 2018). Aguerre et al. (2011) suggested that increased CH_4_ per kg of DMI when FP was increased from 47% to 68% was a result of higher intake of NDF associated with forage rich diets. Therefore, it is likely that increases in NDF intake for the HFP and FOR groups could have contributed to higher CH_4_ yield and intensity observed in the current study. Results here, alongside previous investigations, suggest that increasing concentrate proportion in the diets of dairy cows could be an appropriate strategy to mitigate CH_4_ emissions through increasing DMI and reducing emissions per kg of DMI, milk yield and ECM (Olijhoek et al., 2018). Regression analysis supported this, suggesting that CH_4_ yield might continue to increase between FP 15% to75%, after which, CH_4_ yield reduced whilst CH_4_ intensity showed linear increases as FP increased. The result indicates an increase in CH_4_ intensity by 2.3 g/kg with each increase in FP by 10%. Although, results from REML analysis report similar values between LFP and MFP for CH_4_ yield (CH_4_/DMI) and intensity (CH_4_/milk yield and ECM). This suggests that, supplementing the diet with concentrates between 90% and 70% might not result in any further reduction in these parameters compared to a diet containing concentrates between 70% and 41%. Furthermore, it is important to consider the wider aspect of dairy production; increasing concentrates would not only increase farm production costs, but also represent feed – food competition (Gaudaré et al., 2021).

### 4.2 Effect of breed

There were no differences in intake measurements between breeds, unlike previous investigations into breed, such as McClearn et al. (2022) who reported that HF cows had significantly higher intakes than 3-way crossbred NR × J × HF cows. Olijhoek et al. (2022) similarly found that HF cows had higher feed intakes compared to Jersey cows. It is likely that because the HF cows in the present database originate from the AFBI herd, the genetics are more suitable for a grazing system and therefore do not represent the highest-yielding Holstein cows used in other studies (that would also require and achieve higher DMI).

Breeding goals have historically focused on increasing milk yield and the HF breed is traditionally used in the UK dairy industry for their high yielding capabilities under appropriate, more intensive, management systems (Butler, 2014; Davis et al., 2020). However, the competition for natural resources, particularly that of human edible concentrates (Hayes et al., 2013) has led to the increased adoption of low input dairy farming (characterised by minimal concentrate feeding and the increased utilization of conserved forages or grazing (Scollan et al., 2017)). Subsequently there is interest in crossbreeding and the use of alternative (to HF) breeds with the intention to improve robustness and fertility and reduce labour and production costs (Davis et al., 2020; Vance et al., 2013). Therefore, the concept of feed efficiency in alternative breeds and crossbred animals has also become increasingly valuable when considering breeding goals and management of lower input and pasture-based dairy production systems.

The current study found differences in RFI between breeds with NR cows consuming 0.51 kg/d less compared to NR × HF cows which consumed 0.50 kg/d more than their requirements for maintenance and lactation whilst HF, J × HF and NR cows did not differ from each other. Investigations have suggested that intakes of forage may influence RFI (Ben Meir et al., 2019) but considering the similarities in FP (% DMI) and intakes of NDF, CP and ADF between breeds, it can be assumed that differences in the diet were not the cause of variation in RFI between breeds. The current study found that numerically, HF cows produced 0.09, 0.13 and 0.15 kg more milk per kg of DMI than NR, J × HF and NR × HF, respectively. Findings of higher feed efficiency in HF cows compared to alternative breeds agrees with other studies and could be due to their ability to divert MEI toward milk production over body tissues as suggested by Yan et al. (2006); also reporting higher efficiency calculated at milk energy per kg of MEI for HF compared to NR cows (Yan et al., 2006). Multivariate analysis supports these findings demonstrating negative association between NR cows and feed efficiency parameters. Notably, the present study included data that were collected over 18 years and the genetic potential for yield and efficiency of a breed would have been improved within this time period; for example, in the UK, the average HF milk production has increased by 4500 to 9500 kg from 1980 to recent years (AHDB, 2024).

The current study also observed differences in CH_4_ yield (CH_4_/DMI) between breeds in agreement with previous investigations (Olijhoek et al., 2018). The current study found no differences in CH_4_ yield between HF and J × HF cows, but NR cows produced 1.8 g more CH_4_ per kg of DMI consumed. Results from RDA also suggest positive correlations between NR genetics and increasing CH_4_ yield and intensity parameters. Olijhoek et al. (2018) attributed differences in CH_4_ yield between HF and Jersey cows to differing fermentation pathways which favour H_2_ production over consumption, possibly because of differences in rumen microbial community. However, in contrast to this study Olijhoek et al. (2018) observed that HF cows produced less CH_4_ per kg of DMI compared with Jersey cows when fed 68% DM as forage (32.4 vs 32.5 g CH_4_ per kg of DMI, respectively) and when fed 39% of DM (24.5 vs 27.9 g CH_4_ per kg of DMI, respectively); which further illustrates that the relative reduction on CH_4_ per kg of DMI has also been higher for HF cows when moving towards a lower concentrate diet. It is possible that this might have also been the case for breeds assessed in the current study, although rumen microbiome was not investigated here.

## 5 Conclusions

Increasing dietary FP in dairy systems can safeguard against increased production costs, price volatility, and concerns around food-feed competition and environmental footprint of concentrate feeds. Identifying an optimum forage feeding level in different dairy production systems can have economic and environmental benefits. The present study showed that a reduction in dietary FP from 60% to 87% to 30% to 59% (DM basis), improved productivity (milk and ECM yield) and feed efficiency (milk yield/DMI, ECM/DMI and milk energy/DMI) and reduced CH_4_ yield and intensity. These results can be attributed to the lower NDF and ADF content and higher ME content of diets containing less forage and the subsequent increase in DMI. However, a further reduction in dietary forage proportion to 10% to 30% forage (DM basis) did not result in further improvements in productivity, efficiency or CH_4_ yield or intensity and could negatively affect profitability, given the higher cost of concentrate feed compared with forage. Feed efficiency was similar between diets with 60% to 87% and 100% FP which confirms that it may be economically beneficial for pasture-based low-input systems, to adopt a high-forage diet (by also ensuring animal nutritional requirements are met). The present study also suggests that, when compared with HF or HF × J crosses, the NR cows had the highest RFI, numerically lower milk yield/DMI; whilst multivariate analysis showed NR genetics were negatively associated with productivity and efficiency parameters and positively associated with CH_4_ yield and intensity.

## CRediT authorship contribution statement

**Sabrina Ormston:** Writing – review & editing, Writing – original draft, Visualization, Validation, Methodology, Investigation, Formal analysis, Data curation, Conceptualization. **Tianhai Yan:** Writing – review & editing, Writing – original draft, Visualization, Validation, Supervision, Resources, Project administration, Methodology, Investigation, Funding acquisition, Data curation, Conceptualization. **Xianjiang Chen:** Writing – review & editing, Methodology, Investigation, Data curation. **Alan W. Gordon:** Writing – review & editing, Software, Methodology, Formal analysis. **Katerina Theodoridou:** Writing – review & editing, Supervision. **Sharon Huws:** Writing – review & editing, Supervision. **Sokratis Stergiadis:** Writing – review & editing, Writing – original draft, Visualization, Supervision, Project administration, Methodology, Funding acquisition, Data curation, Conceptualization.

## Data availability

The dataset supporting the conclusions of this article is available on request from the corresponding authors.

## Declaration of competing interest

We declare that we have no financial and personal relationships with other people or organizations that can inappropriately influence our work, and there is no professional or other personal interest of any nature or kind in any product, service and/or company that could be construed as influencing the content of this paper.
