# Supplementary material for: Impact of dietary forage proportion and crossbreeding on feed efficiency and methane emissions in lactating dairy cows
Source: Anim Nutr. 2024 Dec 28;20:419–29. doi: 10.1016/j.aninu.2024.08.011 (PMC11875188; doi:10.1016/j.aninu.2024.08.011)
Supplement: Multimedia component 1 [file mmc1.docx]

## Appendix 1

References of data sources used in the present study are presented below.

Agnew, R. E. 2001. Agri-Food and Biosciences Institute, Hillsborough, UK. Personal communication.

Agnew, R. E., T. Yan, and F. J. Gordon. 1998. Nutrition of the high genetic merit dairy cow-energy metabolism studies. Pages 181–208 in Recent Advances in Animal Nutrition. P. C. Garnsworth and J. Wiseman. ed. Nottingham Univ. Press, Nottingham, UK.

Carrick, I. M., D. C. Patterson, F. J. Gordon, and C. S. Mayne. 1996. The effect of quality and level of protein on the performance of dairy cattle of differing genetic merits. J. Anim. Sci. 62:642.

Cushnahan, A., C. S. Mayne, and E. F. Unsworth. 1995. Effects of ensilage of grass on performance and nutrient utilisation by dairy cattle. 2. Nutrient metabolism and rumen fermentation. J. Anim. Sci. 60:347–359.

Ferris, C. P. 1997. Agri-Food and Biosciences Institute, Hillsborough, UK. personal communication. Ferris, C. P. 2010. Agri-Food and Biosciences Institute, Hillsborough, UK. personal communication.

Ferris, C. P., F. J. Gordon, D. C. Patterson, M. G. Porter, and T. Yan. 1999. The effect of genetic merit and concentrate proportion in the diet on nutrient utilisation by lactating dairy cows. J. Agric. Sci. 132:483–490.

Ferris, C. P., M. A. McCoy, S. D. Lennox, D. C. Catney, and F. J. Gordon. 2001. Nurtient utilization and energy balance associated with two contrasting winter milk production systems for high genetic merit autumn calving dairy cows. Ir. J. Agric. Food Res. 41:55–70.

Ferris, C. P., D. C. Patterson, F. J. Gordon, and D. J. Kilpatrick. 2003. The effect of concentrate feed level on the response of lactating dairy cows to a constant proportion of fodder beet inclusion in a grass silage-based diet. Grass Forage Sci. 57:17–27.

Gordon, F. J., D. C. Patterson, M. G. Porter, and E. F. Unsworth. 2000. The effect of degree of grass wilting prior to ensiling on performance and energy utilisation by lactating dairy cattle. Livest. Prod. Sci. 64:291–294.

Gordon, F. J., D. C. Patterson, T. Yan, M. G. Porter, C. S. Mayne, and E. F. Unsworth. 1995a. The influence of genetic index for milk production on the response to complete diet feeding and the utilisation of energy and nitrogen. J. Anim. Sci. 61:199–210.

Gordon, F. J., M. G. Porter, C. S. Mayne, E. F. Unsworth, and D. J. Kilpatrick. 1995b. The effect of forage digestibility and type of concentrate on nutrient utilisation for lactating dairy cattle. J. Dairy Res. 62:15–27.

Keady, T. W. J., and C. S. Mayne. 1998. The effects of concentrate energy source on silage feeding behaviour and energy utilisation by lactating dairy cows offered grass silages with different intake characteristics. J. Anim. Sci. 67:225–236.

Kirkland, R. M., and F. J. Gordon. 1999. The metabolisable energy requirement for maintenance and the efficiency of use of metabolisable energy for lactation and tissue gain in dairy cows offered a straw/concentrate ration. Livest. Prod. Sci. 61:23–31.

Kirkland, R. M., and F. J. Gordon. 2001. The effects of stage of lactation on the partitioning of, and responses to changes in, metabolisable energy intake in lactating dairy cows. Livest. Prod. Sci. 72:213–224.

Kirkland, R. M., T. Yan, R. E. Agnew, and F. J. Gordon. 2002. Efficiency of use of body tissue energy for milk production in lactating dairy cows. Livest. Prod. Sci. 73:131–138.

Law, R. A., F. J. Young, D. C. Patterson, D. J. Kilpatrick, A. R. G. Wylie, and C. S. Mayne. 2009. Effect of dietary protein content on animal production and blood metabolites of dairy cows during lactation. J. Dairy Sci. 92:1001–1012.

Mayne, C. S. 1995. Agri-Food and Biosciences Institute, Hillsborough, UK. Personal communication.

McCourt, A. R., T. Yan, and C. S. Mayne. 2007. Effect of forage type on methane production from dairy cows. Page 48 in Proc. British Society of Animal Science, Nottingham Univ., Nottingham, UK.

McCourt, A. R., T. Yan, and S. Mayne. 2008. Validation of the sulphur hexafluoride (SF6) tracer technique as a method of predicting methane output from dairy cows. Page 120 in Proc. Br. Soc. Anim. Sci., Nottingham Univ., Nottingham, UK.

McIlmoyle, D. G. 2001. The effect of dietary inclusion of fodder beet on feed utilization and performance of dairy cattle. PhD Thesis. Queen’s Univ., Belfast, UK.

Muñoz, C., T. Yan, D. A. Wills, S. Murray, and A. W. Gordon. 2012. Comparison of the sulfur hexafluoride tracer and respiration chamber techniques for estimating methane emissions and correction for rectum methane output from dairy cows. J. Dairy Sci. 95:3139– 3148.

Patterson, D. C., and D. Kilpatrick. 2009. Effects of feeding forage maize and whole crop silages on the performance of dairy cows offered two qualities of grass silage. Accessed Sep. 24, 2014. http://www.agrisearch.org/attachments/article/120/Alternative_Forages_-_Lo_Res.pdf.

Wylie, A. R. G., S. Woods, A. F. Carson, and M. McCoy. 2008. Periprandial changes in metabolite and metabolic hormone concentrations in high-genetic-merit dairy heifers and their relationship to energy balance in early lactation. J. Dairy Sci. 91:577–586.

Xue, B., T. Yan, C. F. Ferris, and C. S. Mayne. 2011. Milk production and energy efficiency of Holstein and Jersey-Holstein crossbred dairy cows offered diets containing grass silage. J. Dairy Sci. 94:1455–1464.

Yan, T., F. J. Gordon, C. P. Ferris, R. E. Agnew, M. G. Porter, and D. C. Patterson. 1997. The fasting heat production and effect of lactation on energy utilisation by dairy cows offered forage-based diets. Livest. Prod. Sci. 52:177–186.

Yan, T., C. S. Mayne, T. W. J. Keady, and R. E. Agnew. 2006. Effects of dairy cow genotype (Holstein-Friesian versus Norwegian) with two planes of nutrition on energy partitioning between milk and body tissue. J. Dairy Sci. 89:1031–1042.

Yan, T., D. C. Patterson, F. J. Gordon, and M. G. Porter. 1996. The effects of wilting of grass prior to ensiling on the response to bacterial inoculation. 1. Silage fermentation and nutrient utilisation over three harvests. J. Anim. Sci. 62:405–417.

Yan, T., F. J. Young, D. C. Patterson, and C. S. Mayne. 2009. Effects of dietary protein concentration on energetic efficiency and methane emission in lactating dairy cows. Page 42 in Proc. Br. Soc. Anim. Sci., Nottingham Univ., Nottingham, UK.
